# Rituximab for treatment of non-infectious and non-malignant orbital inflammatory disease

**DOI:** 10.1186/s12348-021-00253-3

**Published:** 2021-08-27

**Authors:** Caleb C. Ng, Aileen Sy, Emmett T. Cunningham

**Affiliations:** 1 Department of Ophthalmology, California Pacific Medical Center, San Francisco, CA USA; 2 West Coast Retina Medical Group, 1445 Bush Street, San Francisco, CA 94109 USA; 3 Department of Ophthalmology, Kaiser Permanente Santa Clara, California, USA; 4 Department of Ophthalmology, Stanford University School of Medicine, Stanford, CA USA; 5 Francis I. Proctor Foundation, UCSF School of Medicine, San Francisco, CA USA

**Keywords:** Orbital pseudotumor, Rituxan

## Abstract

**Purpose:**

To provide a comprehensive review of rituximab use for the treatment of non-infectious/non-malignant orbital inflammation.

**Methods:**

Review of literature through January 2021.

**Results:**

Individual data was available for 167 patients with refractory non-infectious/non-malignant orbital inflammation who received treatment with rituximab (RTX). Rituximab was generally utilized as third-line or later treatment (108/149, 72.5%) at a mean of 44.6 months following the diagnosis of orbital inflammation (range = 0 to 360 months; median = 13.7 months). Patients with non-infectious/non-malignant orbital inflammation either received prior treatment with corticosteroids only (27/122, 22.1%), or with one (31/122, 25.4%), two (25/122, 20.5%), or three or more (25/122, 20.5%) corticosteroid-sparing immunosuppressive agents with or without corticosteroids before initiation of RTX treatment. The rheumatologic protocol (two infusions of 1 gram of RTX separated by 14 days) was utilized most frequently (80/144, 55.6%), followed by the oncologic protocol (four weekly infusions of 375 mg/m^2^ RTX; 51/144, 35.4%). Various other off-label regimens were used infrequently (13/144, 9.0%). Rituximab treatments resulted in a positive therapeutic response for the majority of patients with orbital inflammation (146/166, 88.0%). Commonly treated diagnoses included granulomatosis with polyangiitis (99/167, 59.3%), IgG-4 related disease (36/167, 21.6%), and orbital inflammation of indeterminate cause (25/167, 15.0%). No side effects were reported in 83.3% (55/66) of cases. The most common RTX-induced adverse event was an infusion-related temporary exacerbation of orbital disease (4/66, 6.1%), which occurred prior to the routine use of systemic corticosteroids as pre-conditioning.

**Conclusions:**

Overall, RTX appears to be both efficacious and well-tolerated as second- or third-line therapy for patients with non-infectious/non-malignant orbital inflammation.

## Introduction

Non-infectious/non-malignant orbital inflammation accounts for 6–27% of orbital diseases [1, 2]. Possible etiologies include thyroid disease, granulomatosis with polyangiitis (GPA), Churg-Strauss syndrome, polyarteritis nodosa, atypical Cogan syndrome, temporal arteritis, Kawasaki syndrome, Behçet disease, sarcoidosis, Sjögren syndrome, systemic lupus erythematosus (SLE), rheumatoid arthritis, dermatomyositis, and IgG4-related disease [3]. A sizable proportion of cases will remain idiopathic or of indeterminant etiology [2]. High-dose corticosteroids have been utilized as first-line agents due to their availability and efficacy in inducing disease remission [4, 5], but prolonged use is limited by side effects [6]. Patients with disease non-responsive to corticosteroids or requiring long-term immunosuppression have been treated with methotrexate, infliximab, cyclosporine-A, radiotherapy, mycophenolate mofetil, interferon-A, tacrolimus, rituximab (RTX), cyclophosphamide, chlorambucil, leflunomide, and azathioprine, but there is currently no consensus on treatment regimen [7]. The use of RTX has been reported increasingly and with generally good efficacy in 167 cases of refractory non-infectious/non-malignant orbital inflammation, as summarized below.

## Methods

The authors conducted a literature search using the National Library of Medicine’s PubMed database for all English language articles published through January 2021 with the following search terms: “rituximab AND eye”, “rituximab AND orbital inflammation,” “rituximab AND pseudotumor,” and “rituximab AND orbital granuloma.” Relevant references within these articles were also reviewed. Included here were all cases of non-infectious/non-malignant orbital inflammation for which individual case data was available. Articles describing large series of patients in which individual case data was not provided were excluded from the current analysis, but were read for content and citations, and were referenced when appropriate. Individual information on patient age, sex, anatomical localization and cause of disease, prior treatments, time from diagnosis to initiation of RTX, pre and post treatment visual acuities, RTX treatment regimen (dosage and cycles), therapeutic response, additional treatments, duration of follow-up and whether disease recurrence occurred, and adverse events attributed to RTX were collected when available. In this review, a patient was considered to have had a positive therapeutic response to RTX if they achieved disease quiescence, or if the authors subjectively documented improvement in disease severity. Line of therapy was tallied according to the following criteria: first-line – RTX initiated before or at same time as corticosteroids; second-line – RTX initiated after corticosteroids – either alone or with a second more traditional immunosuppressive agent; third-line or greater – RTX initiated after a non-corticosteroid immunosuppressive agent, such as nonbiologic or biologic disease modifying antirheumatic drug, alkylating agents, or intravenous immunoglobulins, with or without corticosteroids. Treatment regimens were classified into either the rheumatologic protocol (two doses of 1000 mg separated by 14 days) [8], the oncologic protocol (four doses of 375 mg/m^2^ weekly) [9], or “other” category for the less commonly utilized dosing protocols. Univariate comparisons were made with two-tailed T-test and nomimal, uncorrected *p*-values were reported.

Rituximab use in thyroid orbitopathy was not included in this review as it has been reviewed previously [10–12]. Use of RTX for non-infectious uveitis and scleritis was summarized in a separate companion review [13].

## Results (Tables 1, 2)

There have been a total of 55 reports describing 167 patients who received treatment with RTX for non-infectious/non-malignant orbital inflammation [14–67]. Overall, there was a male to female ratio of 0.89 to 1, and patients possessed a mean age of 48.0 ± 16.5 years (range = 4 to 86 years; median = 49 years). The anatomical location of orbital inflammation was most commonly described as unspecified orbital inflammation, mass, or granuloma (113/167, 67.6%), followed by orbital inflammation affecting one (22/167, 13.2%), two (21/167, 12.6%), or three or more (11/167, 6.6%) orbital structures. In cases that described the extent of orbital inflammation, 72.2% (39/54) involved the lacrimal gland, 51.9% (28/54) the pre-septal or soft tissues, 50.0% (27/54) one or more extraocular muscles, and 7.4% (4/54) the bones of the orbit. Underlying systemic conditions included GPA (99/167, 28.0%), IgG4-related disease (36/167, 21.6%), indeterminate etiology (25/167, 15.0%), IgG4-related disease and adult orbital xanthogranulomatous disease (AOXGD; 4/167, 2.4%), and one case each (1/167, 0.6%) of AOXGD, GPA and IgG4-related disease, and SLE. Among the 47 cases (28.1%) that reported pre-RTX vision, 51.1% (24/47) had vision better than or equal to 20/40, whereas 27.7% (13/47) had vision worse than or equal to 20/200, and 21.3% (10/47) had vision between 20/40 and 20/200.

**Table 1:** Rituximab use in refractory non-infectious orbital inflammation - summary of comprehensive literature review

Number of Studies	55
Total Patients	167
Age (years)	Mean 48.0±16.5 Median 49 Range 4-86
Gender	0.89:1 (M:F)
Ocular Condition Treated with Rituximab (Ratio, %)	Unspecified Orbital mass/granuloma (71/167, 42.5%); Mass affecting one orbital structure (10/167, 6.0%); Mass affecting two orbital structures (7/167, 4.2%); Mass affecting three or more structures (1/167, 0.6%); Unspecified orbital inflammation (42/167, 25.1%); inflammation affecting one orbital structure (12/167, 7.2%); inflammation affecting two orbital structures (14/167, 8.4%); inflammation affecting three or more orbital structures (10/167, 6.0%) Involved orbital structures when reported: Lacrimal gland (39/54, 72.2%); Extraocular muscles (27/54, 50.0%); Preseptal/soft tissues (28/54, 51.9%); Bone (4/54, 7.4%)
Underlying Systemic Condition (Ratio, %)	GPA (99/167, 59.3%) [8, 9, 14–33]; IgG4-related disease (36/167, 21.6%) [34–48]; Idiopathic orbital inflammation (20/167, 12.0%) [18, 23, 34, 49–57]; Idiopathic sclerosing orbital inflammation (5/167, 3.0%) [58–60]; IgG4-related disease and AOXGD (4/167, 2.4%) [61–64]; AOXGD (1/167, 0.6%) [65]; GPA and IgG4-related disease (1/167, 0.6%) [66]; SLE (1/167, 0.6%) [67]
Treatment Prior to Rituximab (Ratio, %)	None (14/122, 11.5%); Corticosteroid only (27/122, 22.1%); 1 Steroid sparing agent (31/122, 25.4%); 2 Steroid sparing agents (25/122, 20.5%); ≥3 Steroid sparing agents (25/122, 20.5%)
Previously Utilized Immunosuppressants (Ratio, %)	Corticosteroid (92/119, 77.3%); Cyclophosphamide (49/119, 41.2%); Methotrexate (44/119, 37.0%); Azathioprine (34/119, 28.6%); Mycophenolate mofetil (20/119, 16.8%); Infliximab (7/119, 5.9%); TNF-inhibitor NOS (6/119, 5.0%); Cyclosporine (3/119, 2.5%); Adalimumab (2/119, 1.7%); Etanercept (2/119, 1.7%); Chlorambucil (1/119, 0.8%); IV immunoglobulin (1/119, 0.8%); Leflunomide (1/119, 0.8%); Tamoxifen (1/119, 0.8%); Indomethacin (1/119, 0.8%); Vedolizumab (1/119, 0.8%)
Line of Therapy (Ratio, %)	First line (14/149, 9.4%); Second line (27/149, 18.1%); Third line or greater (108/149, 72.5%)
Treatment Regimen and Number of Cycles (Ratio, %)	Rheumatologic (80/144, 55.6%); Oncologic (51/144, 35.4%); Other (13/144, 9.0%) 1 treatment cycle: (79/128, 61.7%); 2 treatment cycles: (28/128, 21.9%); ≥3 treatment cycles: (21/128, 16.4%)
Types of Responses (Ratio, %)	Responsive (146/166, 88.0%) - Disease Remission (93/146, 63.7%) - Author report: (53/146, 36.3%) Nonresponsive (20/166, 12.0%)
Incidence of Recurrence (Ratio, %)	38/126, 30.2%
Adverse Events (Ratio, %)	None (55/66, 83.3%); Exacerbation of orbital disease (4/66, 6.1%); Pneumonitis (2/66, 3.0%)^a^; De novo hepatitis B (1/66, 1.5%); Nausea (1/66, 1.5%); Orbital discomfort with infusion (1/66, 1.5%); Itching and breathlessness with infusion (1/66, 1.5%); Fatigue (1/66, 1.5%)

*RTX* rituximab, *M* male, *F* female, *GPA* granulomatosis with polyangiitis, *AOXGD* adult onset xanthogranulomatous disease, *NOS* not otherwise specified, *Rheumatologic* Two doses of 1000 mg separated by 14 days, *Oncologic* four doses of 375 mg/m2 weekly, *Other* all other RTX dosing regimens, *TNF* tumor necrosis factor, *First line* RTX initiated before or as same time as corticosteroids, *Second line* RTX initiated after corticosteroids, *Third line* RTX initiated after corticosteroids and another agent, such as nonbiologic or biologic disease modifying antirheumatic drug, anti-cancer medications, or intravenous immunoglobulins

^a^one patient died from adenovirus pneumonitis

**Table 2:** Clinical characteristics of non-infectious orbital inflammation cases treated with Rituximab

Identified Cause of Orbital Inflammation	Number (Percentage)	Age (years)	Gender (M:F)	Anatomical Localization (Ratio, %)	Time from Diagnosis to Rituximab Treatment (months)	Rituximab Regimen (Ratio, %)	Positive Therapeutic Response (Ratio, %)	Recurrence (Ratio, %)	Adverse Events (Ratio, %)
Granulomatosis with Polyangiitis	99 (59.3%)	Mean: 42.7 ± 15.6 Median: 42 Range: 4–72	0.89:1	Unspecified Orbital mass/granuloma (64/99, 64.6%); Mass affecting one orbital structure (2/99, 2.0%); Mass affecting three or more structures (1/99, 1.0%); Unspecified orbital inflammation (31/99, 31.3%); Inflammation affecting one orbital structure (1/99, 1.0%) Involved orbital structures when reported: Lacrimal gland (2/4, 50.0%); Extraocular muscles (2/4, 50.0%); Preseptal/soft tissues (2/4, 50.0%)	Mean: 59.4 ± 73.8 Median: 30.8 Range: 0–240	Rheumatologic (51/96, 53.1%); Oncologic (42/96, 43.8%); Other (3/96, 3.1%) 1 treatment cycle: (50/79, 63.3%); 2 treatment cycles: (18/79, 22.8%); > 2 treatment cycles: (11/79, 13.9%) range: 1–8 cycles	84/99, 84.8%	23/74, 31.1%	None (32/38, 84.2%); Exacerbation of disease (3/38, 7.9%); De novo hepatitis (1/38, 2.6%); Nausea (1/38, 2.6%); Adenovirus pneumonitis (1/38, 2.6%)^a^
IgG4-related disease	36 (21.6%)	Mean: 54.8 ± 14.7 Median: 54 Range: 12–83	1 to 0.80	Mass affecting one orbital structure (5/36, 13.9%); Mass affecting two orbital structures (4/36, 11.1%); Unspecified orbital inflammation (3/36, 8.3%); Inflammation affecting one orbital structure (7/36, 19.4%); Inflammation affecting two orbital structures (11/36, 30.6%); Inflammation affecting three or more orbital structures (6/36, 16.7%) Involved orbital structures when reported: Lacrimal gland (23/33, 69.7%); Extraocular muscles (16/33, 48.5%); Preseptal/soft tissues (18/33, 54.5%) Bone (3/33, 9.1%)	Mean: 51.8 ± 88.5 Median: 20.5 Range: 0–360	Rheumatologic (11/19, 57.9%); Oncologic (4/19, 21.1%); Other (4/19, 21.1%) 1 treatment cycle: (10/19, 52.6%); 2 treatment cycles: (2/19, 10.5%); > 2 treatment cycles: (7/19, 36.8%) range: 1–19 cycles	33/35, 94.3%	11/28, 39.3%	None (15/17, 88.2%); Fatigue (1/17, 5.9%); Orbital discomfort with infusion (1/17, 5.9%)
IgG4-related disease and Adult orbital xanthogranulomatous disease	4 (2.4%)	Mean: 51.0 Median: 53.5 Range: 36–61	1 to 0.33	Inflammation affecting one orbital structure (1/4, 25.0%) Inflammation affecting two orbital structures (1/4, 25.0%) Inflammation affecting three or more orbital structures (2/4, 50.0%) Involved orbital structures when reported: Lacrimal gland (3/4, 75.0%); Extraocular muscles (2/4, 50.0%); Preseptal/soft tissues (4/4, 100%)	NR	Rheumatologic (3/3, 100%) 1 cycle: (2/4, 50.0%) 2 cycles: (2/4, 50.0%)	4/4, 100%	0/4, 0%	None (1/1, 100%)
Other^b^	3 (1.8%)	Mean: 53.7 Median: 51 Range: 49–61	0.50:1	Unspecified orbital inflammation (1/3, 33.3%) Orbital inflammation affecting two orbital structures (2/3, 33.3%) Involved orbital structures when reported: Lacrimal gland (2/2, 100%) Extraocular muscles (2/2, 100%)	0^c^	Rheumatologic (2/3, 66.7%); Other (1/3, 33.3%) 1 treatment cycle: (1/3, 33.3%); 2 treatment cycles: (1/3, 33.3%); > 2 treatment cycles: (1/3, 33.3%)	3/3, 100%	0/3, 0%	None (1/1100%)
Indeterminate	25 (15.0%)	Mean: 48.0 ± 19.4 Median: 46 Range: 7–86	0.44:1	Unspecified Orbital mass/granuloma (6/25, 24.0%); Mass affecting one orbital structure (4/25, 16.0%); Mass affecting two orbital structures (2/25, 8.0%); Unspecified orbital inflammation (7/25, 28.0%); Inflammation affecting one orbital structure (3/25, 12.0%) Inflammation affecting two orbital structures (1/25, 4.0%) Inflammation affecting three or more orbital structures (2/25, 8.0%) Involved orbital structures when reported: Lacrimal gland (9/12, 75.0%); Extraocular muscles (5/12, 41.7%); Preseptal/soft tissues (4/12, 33.3%) Bone (1/12, 8.3%)	Mean: 9.9 ± 10.8 Median: 5.0 Range: 1–30	Rheumatologic (12/23, 52.2%); Oncologic (6/23, 26.1%); 4 weekly 10 mg intraorbital injections (3/23, 13.0%); Other (2/23, 8.7%); 1 treatment cycle: (16/23, 69.6%); 2 treatment cycles: (4/23, 17.4%); > 2 treatment cycles: (2/23, 8.7%); range: 1–6 cycles	22/25, 88.0%	6/19, 31.6%	None (6/9, 66.7%); Interstitial pneumonitis (1/9, 11.1%); Exacerbation of orbital disease (1/9, 11.1%); Itching and breathlessness following infusion (1/9, 11.1%)

*M* male, *F* female, *NR* not reported, *Rheumatologic* Two doses of 1000 mg separated by 14 days, *Oncologic* four doses of 375 mg/m2 weekly, *Other* all other RTX dosing regimens

^a^ = patient died from adenovirus pneumonitis

^b^ = includes one case each of systemic lupus erythematosus, concurrent IgG4-related disease and GPA, and necrobiotic xanthogranuloma

^c^ = 2/2 patients received RTX as first line therapy

Among the 166 (99.4%) of patients with documented clinical response, 88.0% (146/166) were responsive to RTX, with 63.7% (93/146) having disease remission and 36.3% (53/146) showing disease improvement or stability based on author report. In contrast, 12.0% (20/166) of patients were described as having treatment failure with RTX. Longitudinal data was available for 126 (86.3%) of those with positive response, revealing that one or more disease recurrences eventually developed in 30.2% (38/126), with interval to first disease relapse occurring at a mean of 21.0 months (range = 2 to 72 months; median = 18.0 months). Details of individualized treatment regimens following uncomplicated RTX therapy was available for 61.4% (54/88) of responders who experienced disease remission without recurrence, showing that 48.1% (26/54) achieving drug-free remission at mean follow up of 28.4 months (range = 4 to 120; median = 14.5 months), 29.6% (16/54) sustaining remission with one or more non-RTX, corticosteroid-sparing systemic immunosuppressive agents at mean follow up of 25.0 months (range = 7 to 60 months; median = 19 months), 13.0% (7/54) maintaining disease quiescence with corticosteroid therapy at mean follow up of 7.9 months (range = 2 to 13 months; median = 9 months), and 9.3% (5/54) still undergoing continued RTX infusions at mean follow up of 34.3 months (range = 12 to 65 months; median = 30 months). Among the patients with reported visual acuities following RTX therapy, 55.6% (25/45) had vision better than or equal to 20/40, 28.9% (13/45) worse than or equal to 20/200, and 15.6% (7/45) between 20/40 and 20/200.

In the 149 cases with a documented treatment history, a total of 72.5% (108/149) received RTX as third-line or later therapy, followed by 18.1% (27/149) as second-line, and 9.4% (14/149) as first-line. In the 122 cases with individualized data on prior therapies, 25.4% (31/122) were treated with one and 20.5% each (25/122) with two or three or more corticosteroid sparing agents, 20% (27/122) used corticosteroids alone, and 11.5% (14/122) were treatment naive. Three patients who received RTX as third-line therapy were described as having prior treatment with corticosteroids and one of several possible corticosteroid-sparing agents. Immunosuppressive agents tried prior to RTX included corticosteroids (92/119, 77.3%), cyclophosphamide (49/119, 41.2%), methotrexate (44/119, 37.0%), azathioprine (34/119, 28.6%), mycophenolate mofetil (20/119, 16.8%), infliximab (7/119, 5.9%), unspecified tumor necrosis factor inhibitor (6/119, 5.0%), cyclosporine (3/119, 2.5%), adalimumab (2/119, 1.7%), etanercept (2/119, 1.7%), chlorambucil (1/119, 0.8%), intravenous immunoglobulin (1/119, 0.8%), leflunomide (1/119, 0.8%), tamoxifen (1/119, 0.8%), indomethacin (1/119, 0.8%), and vedolizumab (1/119, 0.8%). Prior adjunctive radiotherapy was performed on three patients (3/119, 2.5%). The mean time from diagnosis to RTX use was 44.6 ± 71.7 months (*n* = 54; range = 0 to 360 months; median = 13.7 months).

Description of RTX treatment regimens was available for 144 (86.2%) cases, with 55.6% (80/144) receiving the rheumatologic protocol, 35.4% (51/144) receiving the oncologic protocol, and 9.0% (13/144) receiving a variety of uncommon off-label protocols. The number of treatment cycles was reported in 128 subjects (76.6%), with 79 (61.7%) receiving 1 cycle, 28 (21.9%) 2 cycles, and 21 (16.4%) three or more cycles. Among the 79 patients given 1 cycle, 57 (72.2%) included individual information on response to therapy and 36 (45.6%) data regarding recurrences, showing a positive therapeutic response for 88.0% (49/57), no observed response for 12.0% (8/57), sustained disease remission in 88.9% (32/36) at mean follow up of 12.8 months (*n* = 24, range = 2 to 36 months; median = 12 months), and disease recurrence in 11.1% (4/36) at a median of 11 months (*n* = 4; range = 2 to 24 months). Of the 32 cases with sustained remission after 1 cycle, information on post-RTX therapy was available for 24 (75.0%), showing that 41.7% (10/24) were drug free at mean follow up of 10.9 months (*n* = 10; range = 4 to 24 months; median = 12 months), 33.3% (8/24) maintained use of non-RTX immunosuppressants with or without systemic corticosteroids, and 25.0% (6/24) continued systemic corticosteroids alone. Among the 28 patients given two RTX cycles, 24 (85.7%) included individual information on response to therapy and disease recurrence, showing a positive therapeutic response for 100%, sustained disease remission in 63.5% (15/24) at mean follow up of 29.0 months (*n* = 9; range = 4 to 108 months; median = 13.5 months), and disease recurrence in 37.5% (9/24) at a mean of 26.2 months following initial RTX treatment (*n* = 5; range = 12 to 72 months; median = 16 months). Of the 15 cases with sustained remission after two RTX cycles, 60.0% (9/15) were drug free at mean follow up of 33.8 months (*n* = 9; range = 6 to 108 months; median = 15.0 months), 26.7% (4/15) maintained non-RTX immunosuppressants with or without systemic corticosteroids, 6.7% (1/15) continued systemic corticosteroids alone, and another 6.7% (1/15) was scheduled for further RTX treatments. For the nine patients who received 2 cycles and had disease relapse, six (66.7%) received their second course to treat relapse of orbital inflammation that occurred at mean interval of 26.2 months following initial RTX treatment (*n* = 5; range = 12 to 72 months; median = 16 months); the interval between cycles and time until disease recurrence was not reported for the other three cases. Among the 21 patients given three or more cycles, 19 (90.5%) had available individual information on response to therapy and disease recurrence, showing a positive therapeutic response for 94.7% (18/19), no observed response for 5.3% (1/19), sustained disease remission in 83.3% (15/18) at mean follow up of 43.5 months (*n* = 12; range = 12 to 120 months; median = 37 months), and first disease recurrence in 16.7% (3/18) all at 24 months following initiation of RTX. Of the 15 cases with sustained remission after three or more cycles, information on ongoing therapy was available for 12 (75.0%), showing drug-free remission for three (25.0%) patients at a median of 48 months (range = 24 to 120 months), ongoing RTX treatment for four (33.3%), and ongoing non-RTX, corticosteroid-sparing immunosuppressants for another four (33.3%). Patients given two or more RTX cycles were treated at varying intervals: 2 months (2/20, 10.0%) [40, 65], 3 months (1/20, 5.0%) [40], 4 months (1/20, 5.0%) [52], 6 months (10/20, 50.0%) [16, 20, 22, 31, 35, 45, 53, 59, 64, 67], 12 months (1/20, 5.0%) [24], 13 to 18 months (3/20, 15.0%) [15], and 6 years (1/20, 5.0%) [24]. One patient (1/20, 5.0%) received additional RTX cycles at increasing intervals from 3 to 6 months [40]. The patient who received a second RTX cycle at 4 months was being treated for active disease, whereas those who received additional RTX doses at intervals of 12 or more months were treated for disease recurrence. Otherwise, the rationale of designated treatment intervals and indications for retreatment were either unclear or not reported.

Information related to adverse events was provided for 66 subjects (39.5%). A total of 83.3% (55/66) of patients who received RTX treatments for non-infectious/non-malignant orbital inflammation reported no adverse events. Four cases (4/66, 6.1%) experienced orbital disease exacerbation [23, 30, 33], all prior to the routine use of systemic corticosteroids as pre-conditioning, and there was one case each (1/66, 1.5%) of interstitial pneumonitis [53], severe adenovirus pneumonitis leading to death [15], de novo hepatitis B [24], orbital discomfort with infusion [40], itching and breathlessness with infusion [50], nausea [18], and fatigue [40].

### Granulomatosis with Polyangiitis (GPA)

Patients with GPA accounted for 59.3% (99/167) of the overall cohort with non-infectious/non-malignant orbital inflammation who received treatment with RTX. There was a 0.89 to 1 male to female ratio, and mean age at time of treatment was 42.7 years (range = 4 to 72 years; median = 42.0 years). The mean interval from diagnosis of GPA to initiation of RTX treatment was 59.4 months (range = 0 to 240 months; median = 30.8 months). Details regarding the treatment protocol were provided for 96 (97.0%) cases, showing that 53.1% (51/96) received the rheumatologic protocol, 43.8% (42/96) the oncologic protocol, and 3.1% (3/96) with various uncommon off-label treatment regimens. All cases had documented treatment outcomes, with 84.8% (84/99) showing response to RTX, and 15.2% (15/99) experiencing no improvement or further worsening of their orbital inflammation. Of the 84 cases with a positive therapeutic response, 51.2% (43/84) exhibited disease remission, while 48.8% (41/84) were reported to have clinical improvement. Longitudinal data was available for 74 (88.1%) of those responsive to RTX and showed a 31.1% (23/74) rate of eventual disease relapse. Information regarding adverse events was available for 38 (38.4%) of patients. The most common complication was infusion related exacerbation of orbital disease (3/38, 7.9%) that resolved with systemic corticosteroids; all affected patients later experienced remission of orbital inflammation [47, 48, 51]. One case each (1/38, 2.6%) of de novo hepatitis [24], nausea [18], and adenovirus pneumonitis that resulted in death [15] was also described.

### IgG4-related disease

In total, 21.6% of patients (36/167) with non-infectious/non-malignant orbital inflammation were treated with RTX for IgG4-related disease. These patients had a male to female ratio of 1 to 0.80 and mean age of 54.8 years (range = 12 to 83 years; median 54.0 years) when RTX treatment began. The mean interval from diagnosis of IgG4-related orbital inflammation to initiation of RTX treatment was 51.8 months (range = 0 to 360 months; median = 20.5 months). Details regarding the treatment protocol were provided in 19 subjects, including 57.9% (11/19) treated with the rheumatologic protocol, 21.1% (4/19) with the oncologic protocol, and another 21.1% (4/19) with various other uncommon off-label regimens. Treatment outcomes were available for 35 (97.2%) patients, showing a 94.3% (33/35) rate of positive response that was described as disease remission in 78.8% (26/33) and clinical improvement in 21.2% (7/33). No response to RTX was noted for 5.7% (2/25) of cases. Long term data was available for 28 (84.8%) responders, and showed eventual disease relapse in 39.3% (11/28). Adverse event information was provided in 17 (47.2%) subjects, including 15 (88.2%) with no reported no adverse events, one (5.9%) with infusion related orbital discomfort [40], and another (5.9%) with fatigue [40].

### Orbital inflammation of indeterminate etiology

A total of 15.0% (25/167) of patients with non-infectious orbital inflammation who received RTX treatment possessed no discernable systemic etiology for their ocular inflammation. Affected patients possessed a male to female ratio of 0.44 to 1, and the mean age at time of RTX therapy was 48.0 years (range = 7 to 86 years; median = 49.0 years). Details of the RTX treatment was available for 23 (92.0%) subjects, showing that 12 (52.2%) received the rheumatologic protocol, six (26.1%) the oncologic protocol, three (13.0%) with four weekly 10 mg intraorbital RTX injections, and two (8.7%) with other uncommon off-label regimens. A positive therapeutic response was reported in 88.0% (22/25) of patients, and described as remission in 86.4% (19/22) and clinical improvement in 13.6% (3/22). Longitudinal information was available for 19 (86.4%) responders, and showed a 31.6% (6/19) rate of eventual disease recurrence. Presence of adverse events was reported for nine (36.0%) subjects, with six (66.7%) having uncomplicated treatments, and one case each (1/9, 11.1%) developing infusion related orbital disease exacerbation [23], infusion related itching and breathlessness [50], and interstitial pneumonitis [53].

## Discussion

Rituximab, a fully humanized monoclonal anti-CD20 antibody, has increasingly been utilized as a safe and efficacious treatment of refractory, non-infectious/non-malignant orbital inflammation arising from GPA, IgG4-related disease, and indeterminate etiologies. Rituximab was used as a third-line or later treatment in nearly three-quarters of patients with non-infectious orbital inflammation who had been unresponsive to corticosteroid or other corticosteroid-sparing immunosuppressants, with treatment generally initiated nearly 4 years following initial diagnosis. Half of these patients received the rheumatologic protocol, two fifths the oncologic protocol, and nearly one fifth various off-label dosing regimens. More than three fifths of treated patients received only 1 cycle of RTX, while one fifth received 2 cycles, and one sixth three or more cycles. Patients with orbital inflammation had a positive therapeutic response in nearly nine out of 10 cases, with more than three fifths experiencing disease remission or regression, and one sixth reporting generally mild adverse events, the most common of which were infusion reactions.

Use of RTX for treatment of non-infectious/non-malignant orbital inflammation was efficacious for a variety of underlying systemic causes. All of the patients with orbital inflammation secondary to both IgG4-related disease and AOXGD (*n* = 4, 100%) had a positive therapeutic response, while somewhat less efficacy was observed for patients with orbital inflammation attributed to IgG4-related disease (*n* = 35, 94.3%), for those with GPA (*n* = 99, 84.8%) and those with an indeterminate etiology (*n* = 25, 88.0%). Pair-wise differences in rates of positive therapeutic response for the most common underlying causes of non-infectious/non-malignant orbital inflammation did not reach a nominal *p*-value of less than 0.05. The overall efficacy of RTX for treatment of orbital inflammation (88.0%) was comparable to what has been reported for non-infectious uveitis (83.5%) and scleritis (93.3%) [13].

While a variety of RTX dosing regimens and cycles were utilized to treat non-infectious/non-malignant orbital inflammation, the rheumatologic protocol was both most commonly utilized and numerically most efficacious in reducing disease severity, while the overall number of treatment cycles had no discernable impact on outcomes. Compared to patients who received the oncologic protocol, which was the second most common treatment regimen, patients given the rheumatologic protocol had a significantly higher chance of a positive therapeutic response (nominal *p* = < 0.0001, N-1 two-proportion test, two-tailed). The remaining subjects received various other off-label dosing regimens, but the collected data was insufficient to allow for comparison due to limited sample sizes and lack of standardization across the studies. Excluding the difference in proportion of patients who achieved continued disease quiescence following one and two RTX cycles (nominal *p* < 0.015, N-1 two-proportion test, two-tailed), pair-wise comparisons of the rates of positive therapeutic response, sustained disease remission, and drug-free remission between patients given one, two, and three or more cycles of RTX failed to achieve a nominal *p*-value of < 0.05. Patients who received one RTX cycle leading to disease remission and were eventually given a second RTX cycle for disease relapse (*n* = 5; mean of 26.2 months) were categorized into the two-cycle group, which may explain the significant difference in rates of sustained disease remission between the two subgroups. Overall, these findings suggest the rheumatologic protocol could be considered for the treatment of non-infectious/non-malignant orbital inflammation – particularly those patients who fail first-line immunosuppression therapy.

The majority of patients with non-infectious/non-malignant orbital inflammation received RTX as a third-line agent, generally after unsuccessful treatments with corticosteroid and traditional non-corticosteroid immunosuppressive agents. Possible contributing factors for this practice pattern could have been the cost of RTX, a relatively more difficult route of administering RTX, or provider unfamiliarity with RTX as a treatment choice in such patients. Of note, RTX was more likely to be used earlier in the treatment course for patients with IgG4-related disease as compared to patients with GPA. While nearly all patients with orbital inflammation from GPA received RTX as third-line treatment (79/82, 96.3%), those with IgG4-RD received RTX as a third-line agent in just over a third of cases (13/36, 36.1%). In total, 71.4% (10/14) of all cases of orbital inflammation treated with RTX as first-line therapy and 63.0% (17/27) of those given RTX as second-line therapy possessed orbital inflammation attributed to IgG4-related disease alone or concurrently with another etiology. To our knowledge, Plaza et al. first described in 2010 the use of RTX as a first-line immunosuppressant to treat six patients with IgG4-related orbital inflammation, but did not elaborate on their treatment approach [34]. Later, Murakami et al. promptly used RTX to treat a patient with IgG4-related orbital inflammation due to the presence of elevated serum IgG and abnormal lymphoid tissue found on histology [39], and Pomponio et al. used RTX as a first-line agent to treat orbital inflammation from concurrent IgG4-related disease and AOXGD due to the presence of elevated serum plasmablasts and the desire to avoid potential corticosteroid related side effects in a post-menopausal woman [63]. The generally positive outcomes noted here suggest that RTX might be considered earlier in the course of therapy in some patients.

Clinical trials investigating RTX treatment for rheumatologic diseases have found it to be relatively well-tolerated with mild to moderate infusion-related reactions as the most common adverse response [68–70]. The known severe adverse reactions include tumor lysis syndrome, severe mucocutaneous reactions, progressive multifocal leukoencephalopathy, hepatitis B reactivation, infections, cardiac arrhythmias, renal toxicity, and bowel obstruction and perforation [71]. While there have been a large number of reports detailing the efficacy of RTX in treatment of orbital inflammation, less than half (66/167, 39.4%) included information on drug safety, with one-sixth (11/66, 16.7%) of those reported to have experienced an adverse event. According to the common terminology criteria for adverse events, 90.9% (10/11) of reported RTX-associated side effects were grade 1 to 3 reactions. However, one patient (1/11, 9.1%) developed adenovirus pneumonitis 4 months after his second dose of RTX that ultimately caused his death (grade 5). An infusion reaction leading to exacerbation of orbital inflammation, was described early in the treatment course for four patients, three of that later achieved disease remission following RTX treatment. These authors proposed the use of systemic corticosteroids before, during, and after RTX infusions so as to lessen or prevent such inflammatory infusion reactions [36, 59, 65]. Following orbital exacerbations in the first three of four RTX-treated patients, Suhler et al. implemented use high-dose oral prednisone for 3 days before and after RTX infusions, and prevented further inflammatory flares at and after infusion [36]. Overall, the rate of reported adverse events was similar to or lower than those reported in clinical trials, similar to the rates gleaned from a similarly conducted retrospective review of the use of RTX for non-infectious uveitis (23.7%; nominal *p* = 0.28, N-1 two-proportion test, two-tailed) and scleritis (14.5%; nominal *p* = 0.71, N-1 two-proportion test, two-tailed) [13].

The retrospective nature of our analysis was limited by variations in details regarding prior treatment, the definition of positive therapeutic response, treatment protocols, rationale for retreatment, total number of RTX cycles, duration of follow-up and whether disease relapse occurred, and of the occurrence of adverse events. Several larger series provided overall cohort statistics only and so could not be incorporated into our calculations. Finally, the efficacy of the studies reviewed here may not reflect a broader population-based sample of refractory non-infectious/non-malignant orbital inflammation due to referral, selection, treatment, evaluator, and/or publication bias [72]. Safety and adverse events data was explicitly provided for fewer than half of cases.

## Conclusions

Rituximab appears to be an effective and well-tolerated treatment option for patients with non-infectious/non-malignant orbital inflammation. Authors generally favored use of one or two RTX cycles according to the rheumatologic or oncologic dosing. Our findings suggest that the rheumatologic protocol may be superior in inducing disease remission, but similar reported rates of positive response and sustained disease remission were achieved in those given limited and extended treatment courses. Adverse events were reported in about one-sixth of treated patients and tended to be mild to moderate infusion reactions. Overall, the current ophthalmologic literature strongly supports RTX use for non-infectious/non-malignant orbital inflammation refractory to corticosteroid and traditional non-corticosteroid immunosuppressive agents, especially GPA and IgG4-related disease.

## Data Availability

The datasets used and/or analyzed during the current study are available from the corresponding author on reasonable request.

## Abbreviations

AOXGD: Adult orbital xanthogranulomatous disease
GPA: Granulomatosis with polyangiitis
RTX: Rituximab
SLE: Systemic lupus erythematosus

## References

[1] Gordon LK (2006). Orbital inflammatory disease: a diagnostic and therapeutic challenge. Eye.

[2] Shields JA, Shields CL, Scartozzi R (2004). Survey of 1264 patients with orbital tumors and simulating lesions: the 2002 Montgomery lecture, part 1. Ophthalmology.

[3] Mombaerts I, Rose GE, Garrity JA (2016). Orbital inflammation: biopsy first. Survey Ophthalmol.

[4] Leone CR, Lloyd WC (1985). Treatment protocol for orbital inflammatory disease. Ophthalmology.

[5] Yuen SJ, Rubin PA (2003). Idiopathic orbital inflammation: distribution, clinical features, and treatment outcome. Arch Ophthalmol.

[6] Cunningham ET, Wender JD (2010). Practical approach to the use of corticosteroids in patients with uveitis. Can J Ophthalmol.

[7] Espinoza GM (2010). Orbital inflammatory pseudotumors: etiology, differential diagnosis, and management. Curr Rheumatol Rep.

[8] Cheung CM, Murray PI, Savage CO (2005). Successful treatment of Wegener’s granulomatosis associated scleritis with rituximab. Brit J Ophthalmol.

[9] Ahmadi-Simab K, Lamprecht P, Nölle B, Ai M, Gross WL (2005). Successful treatment of refractory anterior scleritis in primary Sjögren’s syndrome with rituximab. Ann Rheum Dis.

[10] Salvi M, Vannucchi G, Beck-Peccoz P (2013). Potential utility of rituximab for Graves’ orbitopathy. J Clin Endocrinol Metab.

[11] Rao R, MacIntosh PW, Yoon MK, Lefebvre DR (2015). Current trends in the management of thyroid eye disease. Curr Opin Ophthalmol.

[12] Minakaran N, Ezra DG (2013). Rituximab for thyroid-associated ophthalmopathy. Cochrane Database Syst Rev.

[13] Ng CC, Sy A, Cunnningham ET Jr (2021) Rituximab for non-infectious uveitis and scleritis – accepted for publication in Journal of ophthalmic inflammation and infection 6/2021. 10.1186/s12348-021-00252-4 PMC8364894 34396463

[14] Aries PM, Hellmich B, Voswinkel J, Both M, Nölle B, Holl-Ulrich K, Lamprecht P, Gross WL (2006). Lack of efficacy of rituximab in Wegener’s granulomatosis with refractory granulomatous manifestations. Ann Rheum Dis.

[15] Seo P, Specks U, Keogh KA (2008). Efficacy of rituximab in limited Wegener’s granulomatosis with refractory granulomatous manifestations. J Rheumatol.

[16] Taylor SR, Salama AD, Joshi L, Pusey CD, Lightman SL (2009). Rituximab is effective in the treatment of refractory ophthalmic Wegener’s granulomatosis. Arthritis Rheumatism.

[17] Ooka S, Maeda A, Ito H, Omata M, Yamada H, Ozaki S (2009). Treatment of refractory retrobulbar granuloma with rituximab in a patient with ANCA-negative Wegener’s granulomatosis: a case report. Mod Rheumatol.

[18] Kurz PA, Suhler EB, Choi D, Rosenbaum JT (2009). Rituximab for treatment of ocular inflammatory disease: a series of four cases. Br J Ophthalmol.

[19] Holle JU, Dubrau C, Herlyn K, Heller M, Ambrosch P, Noelle B, Reinhold-Keller E, Gross WL (2012). Rituximab for refractory granulomatosis with polyangiitis (Wegener's granulomatosis): comparison of efficacy in granulomatous versus vasculitic manifestations. Ann Rheum Dis.

[20] Dalkilic E, Alkis N, Kamali S (2012). Rituximab as a new therapeutic option in granulomatosis with polyangiitis: a report of two cases. Mod Rheumatol.

[21] Baslund B, Wiencke AK, Rasmussen N, Faurschou M, Toft PB (2012). Treatment of orbital inflammation with rituximab in Wegener’s granulomatosis. Clin Exp Rheumatol.

[22] Pelegrin L, Jakob E, Schmidt-Bacher A, Schwenger V, Becker M, Max R, Lorenz HM, Mackensen F (2014). Experiences with rituximab for the treatment of autoimmune diseases with ocular involvement. J Rheumatol.

[23] Suhler EB, Lim LL, Beardsley RM, Giles TR, Pasadhika S, Lee ST, de Saint SA, Butler NJ, Smith JR, Rosenbaum JT (2014). Rituximab therapy for refractory orbital inflammation: results of a phase 1/2, dose-ranging, randomized clinical trial. JAMA Ophthalmol.

[24] Nagafuchi H, Atsumi T, Hatta K, Muso E, Takeno M, Yamada H, Ozaki S (2015). Long-term safety and efficacy of rituximab in 7 Japanese patients with ANCA-associated vasculitis. Mod Rheumatol.

[25] Bitik B, Kılıç L, Küçükşahin O, Şahin K, Tufan A, Karadağ Ö, Pay S, Ateş A, Ucar M, Tutar H, Karaaslan Y (2015). Retro-orbital granuloma associated with granulomatosis with polyangiitis: a series of nine cases. Rheumatol Int.

[26] Joshi L, Tanna A, McAdoo SP, Medjeral-Thomas N, Taylor SR, Sandhu G, Tarzi RM, Pusey CD, Lightman S (2015). Long-term outcomes of rituximab therapy in ocular granulomatosis with polyangiitis: impact on localized and nonlocalized disease. Ophthalmology.

[27] Caçola RL, Morais SA, Carvalho R, Môço R (2016). Case report: bilateral dacryoadenitis as initial presentation of a locally aggressive and unresponsive limited form of orbital granulomatosis with polyangiitis. BMJ Case Rep.

[28] Kenny GM, Holl-Ulrich K, Fulcher T, McElnea E, Kavanagh E, Moriarty H, Mulligan N, Molloy ES, McCarthy GM (2018). Successful reconstruction of an ocular defect resulting from granulomatosis with polyangiitis, following treatment with rituximab. Am J Ophthalmol Case Rep.

[29] Asín MA, Charles P, Rothschild PR, Terrier B, Brézin A, Mouthon L, Guillevin L, Puéchal X (2019). Ocular involvement in granulomatosis with polyangiitis: a single-center cohort study on 63 patients. Autoimmun Rev.

[30] Shih CB, Wang YC, Lai CC (2019). Ocular and orbital exacerbation after rituximab therapy for granulomatosis with polyangiitis. Can J Ophthalmol.

[31] Riancho-Zarrabeitia L, Peiró Callizo E, Drake-Pérez M, García Montesinos B, Terán N, Martínez-Taboada VM (2019). Granulomatosis with polyangiitis with isolated orbital involvement in children: a case report successfully treated with Rituximab and review of literature. Acta Reumatol Port.

[32] Franco J, Lee NG (2020). Granulomatosis with polyangiitis presenting as recurrent, multifocal orbital myositis. Orbit.

[33] Babu K, Dharmanand BG (2020). Worsening of posterior scleritis and orbital pseudotumor in a patient with granulomatosis polyangiitis with rituximab-a case report. Indian J Ophthalmol.

[34] Plaza JA, Garrity JA, Dogan A, Ananthamurthy A, Witzig TE, Salomao DR (2011). Orbital inflammation with IgG4-positive plasma cells: manifestation of IgG4 systemic disease. Arch Ophthalmol.

[35] Khosroshahi A, Bloch DB, Deshpande V, Stone JH (2010). Rituximab therapy leads to rapid decline of serum IgG4 levels and prompt clinical improvement in IgG4-related systemic disease. Arthritis Rheumatism.

[36] Khosroshahi A, Carruthers MN, Deshpande V, Unizony S, Bloch DB, Stone JH (2012). Rituximab for the treatment of IgG4-related disease: lessons from 10 consecutive patients. Medicine.

[37] Wallace ZS, Khosroshahi A, Jakobiec FA, Deshpande V, Hatton MP, Ritter J, Ferry JA, Stone JH (2012). IgG4-related systemic disease as a cause of “idiopathic” orbital inflammation, including orbital myositis, and trigeminal nerve involvement. Surv Ophthalmol.

[38] Sane M, Chelnis J, Kozielski R, Fasiuddin A (2013). Immunoglobulin G4–related sclerosing disease with orbital inflammation in a 12-year-old girl. J Am Assoc Pediatr Ophthalmol Strabismus.

[39] Murakami J, Matsui S, Ishizawa S, Arita K, Wada A, Miyazono T, Hounoki H, Shinoda K, Taki H, Sugiyama T (2013). Recurrence of IgG4-related disease following treatment with rituximab. Mod Rheumatol.

[40] Wu A, Andrew NH, Tsirbas A, Tan P, Gajdatsy A, Selva D (2015). Rituximab for the treatment of IgG4-related orbital disease: experience from five cases. Eye.

[41] Chen TS, Figueira E, Lau OC, McKelvie PA, Smee RI, Dawes LC, Agar A, Wilcsek G, Francis IC (2014). Successful “medical” orbital decompression with adjunctive rituximab for severe visual loss in IgG4-related orbital inflammatory disease with orbital myositis. Ophthalmic Plast Reconstr Surg.

[42] Yu WK, Kao SC, Yang CF, Lee FL, Tsai CC (2015). Ocular adnexal IgG4-related disease: clinical features, outcome, and factors associated with response to systemic steroids. Jpn J Ophthalmol.

[43] Lee CS, Harocopos GJ, Kraus CL, Lee AY, Van Stavern GP, Couch SM, Rao PK (2015). IgG4-associated orbital and ocular inflammation. J Ophthalmic Inflam Infect.

[44] Ebbo M, Patient M, Grados A, Groh M, Desblaches J, Hachulla E, Saadoun D, Audia S, Rigolet A, Terrier B, Perlat A, Guillaud C, Renou F, Bernit E, Costedoat-Chalumeau N, Harlé JR, Schleinitz N (2017). Ophthalmic manifestations in IgG4-related disease: Clinical presentation and response to treatment in a French case-series. Medicine (Baltimore).

[45] Aouidad I, Schneider P, Zmuda M, Gottlieb J, Viguier M (2017). IgG4-related disease with orbital pseudotumors treated with rituximab combined with palpebral surgery. JAMA Dermatol.

[46] Lemaitre S, Esquerda GM, Guardiola AC, Agustin JT, Sanda N, González-Candial M (2018). Colon cancer and IgG4-related disease with orbital inflammation and bilateral optic perineuritis: A case report. Medicine (Baltimore).

[47] Karim AF, Bansie RD, Rombach SM, Paridaens D, Verdijk RM, van Hagen PM, Van Laar JA (2018). The treatment outcomes in IgG4-related disease. Neth J Med.

[48] Queen D, Hedayat AA, Magro C, Geskin LJ (2019). An unusual cause of bilateral orbital swelling: immunoglobulin G4–related orbital disease arising in a patient with ulcerative colitis. JAAD Case Rep.

[49] Schafranski MD (2009). Idiopathic orbital inflammatory disease successfully treated with rituximab. Clin Rheumatol.

[50] Ibrahim I, Barton A, Ibrahim A, Ho P (2012). Idiopathic orbital inflammation successfully treated using rituximab in a patient with rheumatoid arthritis. J Rheumatol.

[51] Shao EH, Karydis A, Gemenetzi M, Taylor SR (2013). Successful corticosteroid-sparing effect of rituximab in the treatment of refractory idiopathic orbital inflammatory disease. Case Rep Ophthalmol.

[52] Savino G, Battendieri R, Siniscalco A, Mandarà E, Mulè A, Petrone G, Traina S, Riso M (2015). Intraorbital injection of rituximab in idiopathic orbital inflammatory syndrome. Rheumatol Int.

[53] Kasi SK, Kim HJ, Basham RP, Cunningham ET, Sy A, Lustig L, Kersten RC (2016). Idiopathic orbital inflammation associated with necrotizing scleritis and temporal bone inflammation. Ophthalmic Plast Reconstr Surg.

[54] Sakano H, Shih CP, Jafari A, DeConde A, Harris JP (2018). Multifocal inflammatory Pseudotumor of the temporal bone, maxillary sinus, and orbit. Otol Neurotol.

[55] Vrcek I, Wotipka E, Somogyi M, Nakra T (2018). Rapid and complete vision loss due to idiopathic orbital inflammation with partial recovery following orbital decompression. Baylor Univ Med Center Proc.

[56] Laban KG, Kalmann R, Leguit RJ, de Keizer B (2019). Zirconium-89-labelled rituximab PET-CT in orbital inflammatory disease. EJNMMI Res.

[57] Artieda JA, Elias IT (2020). Tocilizumab in a case of refractory idiopathic orbital inflammation: 6-year follow-up outcomes. Case Rep Ophthalmol.

[58] On AV, Hirschbein MJ, Williams HJ, Karesh JW (2006). CyberKnife radiosurgery and rituximab in the successful management of sclerosing idiopathic orbital inflammatory disease. Ophthalmic Plast Reconstr Surg.

[59] Abell RG, Patrick A, Rooney KG, McKelvie PA, McNab AA (2015). Complete resolution of idiopathic sclerosing orbital inflammation after treatment with rituximab. Ocul Immunol Inflamm.

[60] Yazici B, Cekic S, Yalcinkaya U, Kilic SS (2020). Rituximab Therapy for Recalcitrant Idiopathic Sclerosing Orbital Inflammation. Ophthalmic Plastic Reconstruct Surg.

[61] Burris CK, Rodriguez ME, Raven ML, Burkat CN, Albert DM (2016). Adult-onset asthma and periocular xanthogranulomas associated with systemic IgG4-related disease. Am J Ophthalmol Case Rep.

[62] Leung KC, Ko P, Ho CH, Ko TC (2018). Adult onset xanthogranuloma associated with IgG4-related disease. Korean J Ophthalmol.

[63] Pomponio G, Olivari D, Mattioli M, Angeletti A, Rossetti G, Goteri G, Gabrielli A (2018). Sustained clinical response after single course of rituximab as firstline monotherapy in adult-onset asthma and periocular xanthogranulomas syndrome associated with IgG4-related disease: A case report. Medicine (Baltimore).

[64] Asproudis I, Kanari M, Ntountas I, Ragos V, Goussia A, Batistatou A, Voulgari PV (2020). Successful treatment with rituximab of IgG4-related disease coexisting with adult-onset asthma and periocular xanthogranuloma. Rheumatol Int.

[65] Sagiv O, Thakar SD, Morrell G, Tetzlaff MT, Esmaeli B (2018). Rituximab monotherapy is effective in treating orbital necrobiotic xanthogranuloma. Ophthal Plast Reconstr Surg.

[66] Della-Torre E, Lanzillotta M, Campochiaro C, Bozzalla E, Bozzolo E, Bandiera A, Bazzigaluppi E, Canevari C, Modorati G, Stone JH, Manfredi A, Doglioni C (2016). Antineutrophil cytoplasmic antibody positivity in IgG4-related disease: A case report and review of the literature. Medicine (Baltimore).

[67] González CM, Montero SR, Pérez RM, Mañosa CP, Feijoo ML, de La Fuente JL (2010). Resistant orbital pseudotumor treated with rituximab in a patient with systemic lupus erythematosus. A case presentation. Reumatol Clín.

[68] van Vollenhoven RF, Emery P, Bingham CO, Keystone EC, Fleischmann R, Furst DE, Macey K, Sweetser M, Kelman A, Rao R (2010). Longterm safety of patients receiving rituximab in rheumatoid arthritis clinical trials. J Rheumatol.

[69] Van Vollenhoven RF, Emery P, Bingham CO, Keystone EC, Fleischmann RM, Furst DE, Tyson N, Collinson N, Lehane PB (2013). Long-term safety of rituximab in rheumatoid arthritis: 9.5-year follow-up of the global clinical trial programme with a focus on adverse events of interest in RA patients. Ann Rheum Dis.

[70] Cohen SB, Emery P, Greenwald MW, Dougados M, Furie RA, Genovese MC, Keystone EC, Loveless JE, Burmester GR, Cravets MW, Hessey EW (2006). Rituximab for rheumatoid arthritis refractory to anti–tumor necrosis factor therapy: results of a multicenter, randomized, double-blind, placebo-controlled, phase III trial evaluating primary efficacy and safety at twenty-four weeks. Arthritis Rheumatism.

[71] Genentech Inc (2010). Rituxan (rituximab) [package insert].

[72] Cunningham ET, Acharya N, Kempen JH, Zierhut M (2015). Design and interpretation of clinic-based studies in uveitis. Ocul Immunol Inflamm.

